# Biological Benchmarks for Adult Bone Mass Proportions in Young Females: A Prospective Longitudinal Analysis

**DOI:** 10.1002/ajhb.70118

**Published:** 2025-08-13

**Authors:** Jodi N. Dowthwaite, Stephanie A. Kliethermes, Tamara A. Scerpella

**Affiliations:** ^1^ Department of Orthopedics and Rehabilitation University of Wisconsin‐Madison Madison Wisconsin USA; ^2^ Department of Orthopedic Surgery SUNY Upstate Medical University Syracuse New York USA

**Keywords:** adolescent, bone mass, DXA, growth, menarche

## Abstract

**Objectives:**

In growing humans, densitometric scans of whole‐body bone mass “less head” are recommended to circumvent the excessive contribution of youths' proportionally larger heads but potentially inflate inter‐scan variation and least significant change due to measurement error. We aimed to determine biological benchmarks for achievement of adult head‐body proportions in a sample of US females.

**Methods:**

Annual whole‐body dual energy x‐ray absorptiometry (DXA) scans tracked growth, maturation, and bone mass accrual in a prospective longitudinal cohort of girls for up to 19 years (baseline age 7–15 years). We used cubic smoothing spline mixed effects models to generate chronological and gynecological age‐based curves for head versus whole‐body bone mass proportions (ratios). Females with ≥ 3 annual scans were included (*n* = 148, age 7–30 years).

**Results:**

Models yielded trajectories extending beyond observed age at peak bone mass for our sample. From age 18 years, “adult” mean of means for head vs. whole‐body bone mass proportions was 0.204 (*n* = 66: 95% confidence interval = 0.198–0.210). Individual proportions stabilized to “adult” mean levels circum‐menarche (*n* = 124: mean = 0.198; 95% confidence interval = 0.194–0.202). The minimum age for 95% confidence intervals overlapping with adult values was 12 years, circum‐peak height velocity (*n* = 120: mean = 0.211; 95% confidence interval = 0.207–0.216).

**Conclusion:**

In US girls with diverse activity exposures, head vs. whole‐body bone mass proportions are “adult” from menarche onward; an “adult” age threshold of 12 years, or age at peak height velocity, may be used in the absence of extreme maturational delay to evaluate whole‐body bone mass including the head.

## Graphical abstract

In the current longitudinal study of bone mineral accrual from childhood into adulthood, US girls with diverse activity exposures demonstrated “adult” head versus whole‐body bone mass proportions after menarche (gynecological age = 0+). After menarche, use of whole‐body BMC avoids potential for inflation of both measurement error and least significant change associated with total body less head assessments (recommended in childhood).

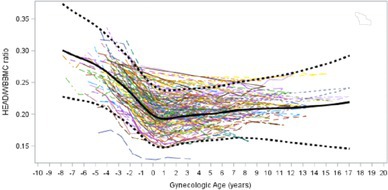

## 1 Introduction

In early life, humans exhibit variable patterns of saltatory growth and stasis that are genetically determined but also responsive to environmental factors such as nutrient availability, energetic demands, and other stressors (Lampl and Schoen 2017). Interactions of these non‐modifiable and modifiable factors affect growth plate metabolic processes to drive changes in long bone growth, body proportions, and height (Lampl and Schoen 2017). Beyond linear growth, genetic and environmental factors shape overarching patterns of physical maturation that vary widely among youths of both sexes. Females exhibit heterogeneous patterns of menarche and menopause timing relative to chronological age, as a function of genetic factors and early life environmental triggers, including nutrition and energetic signaling in utero and during childhood (Kentistou et al. 2024). These genetic factors interact with body weight through central sensors in the hypothalamus, yielding bidirectional relationships with processes of maturation, growth, reproduction, and senescence; the results are observed as broad phenotypic variation in hormone levels, tissue characteristics, and risks of associated diseases across the lifespan (Kentistou et al. 2024).

For both cortical and cancellous bone tissue, variable estrogen levels affect patterns of linear growth and responses to mechanical loading in both sexes during youth (Noirrit‐Esclassan et al. 2021). Interactions of estrogen and mechanical factors drive bone modeling and remodeling to influence site‐ and tissue‐specific bone mass, geometry, and density across axial and appendicular skeletal sites (Noirrit‐Esclassan et al. 2021). In youth, wide variance in maturational patterns and associated estrogen‐loading interactions compound to influence peak bone mass and strength, yielding accumulated disadvantages for females relative to males (Noirrit‐Esclassan et al. 2021). Thus, childhood and adolescence are critical periods for bone structural development, with lasting consequences across the lifespan. Environmentally labile patterns of maturity‐ and site‐specific bone mineral accrual vary widely at sub‐cranial regions of interest in response to physical activity exposure and withdrawal (Scerpella et al. 2016); in contrast, limited variance is observed for skull bone accrual patterns in the approach to peak bone mass (Scerpella et al. 2011). Because the timing of complex linear growth and site‐specific bone accrual patterns varies widely by chronological age, biological age benchmarks are needed to guide skeletal assessment in childhood and adolescence.

Because pediatric bone accrual patterns affect fracture risk during youth and set the stage for fracture risk in adulthood, it is important to monitor bone health across developmental phases. In adults, dual energy x‐ray absorptiometry (DXA) whole‐body bone mineral content (WBBMC, g) may be monitored to summarize “bone mass” across disparate skeletal sites. However, changing head to body proportions from childhood to adulthood make the use of WBBMC problematic. For pediatric studies, the International Society for Clinical Densitometry recommends excluding skull BMC to monitor WBBMC “less head” (sub‐head BMC, SUBBMC, g) (Gordon et al. 2014). SUBBMC assessment is used to blunt the confounding influence of a proportionally larger skull to whole‐body size in children, compared to adult proportions. After infancy, most fractures are not craniofacial; thus, SUBBMC aims to capture bone accrual processes relevant to fracture risk in childhood and adolescence (Naranje et al. 2016; Randsborg et al. 2013). Yet, SUBBMC protocols were designed to monitor youths with congenital or early onset chronic conditions that may affect physical activity, maturation, and/or BMC. Accordingly, this methodology may be less appropriate for monitoring healthy, physically active youths in research and clinical practice related to pediatric exercise science, nutrition, endocrinology, and sports medicine.

Furthermore, during growth, head vs. body size proportions decrease gradually to achieve adult proportions, so there must be a threshold beyond which youth head:body BMC ratios become indistinguishable from adult proportions. This threshold is important, as subdivision during scan analysis adds measurement error (Dowthwaite et al. 2018), likely due to variable head position and/or reference line placement. This unnecessary error may confound serial DXA tracking, cutting or boosting follow‐up SUBBMC relative to prior measurements. When monitoring youth athletes and teens with nutritional concerns, such as relative energy deficiency in sport (REDs), measurement error should be minimized for research interpretation and clinical decision‐making. Reliability and accuracy are paramount for evaluations of youth skeletal responses to exercise, nutrition, and pharmacological interventions. Because the bulk of adult BMC is accrued rapidly during adolescence (Baxter‐Jones et al. 2011; Lappe et al. 2015), valid measurements are critical for timely initiation and cessation of osteogenic interventions across this transition from childhood to adulthood. Ensuring age‐appropriate use of WBBMC during adolescence, instead of SUBBMC, would decrease measurement error and reduce least significant change (LSC) during this important period of bone accrual to reach peak bone mass. However, longitudinal studies have not determined the biological age at which the HEADBMC to WBBMC ratio (HEAD:WB BMC) reaches adult proportions; age 18 years appears to be the default to apply “adult” bone assessment protocols (Gordon et al. 2014).

To determine timing of the transition from pediatric head:whole‐body proportions to stable adult proportions, relevant biological benchmarks should be defined based on longitudinal data encompassing substantial variation in maturational timing and associated physical activity exposure. This approach would yield data that encompasses heterogeneous estrogen levels, energetic stresses, mechanical loads, and associated interactions. Thus, we aimed to determine biological benchmarks for appropriate use of WBBMC monitoring in US female adolescents with diverse physical activity exposures across growth and maturation. We hypothesized that HEAD:WB BMC would reach adult proportions in adolescent females circum‐menarche, earlier than the arbitrary age of legal adulthood (18 years).

## 2 Materials and Methods

For over 20 years, we have used annual whole‐body DXA scans to monitor pediatric bone accrual into adulthood. Research protocols were approved by our Institutional Review Board to comply with the Declaration of Helsinki. At baseline, girls were free from diseases and/or medications affecting bone growth. At enrollment, all participants were minors who provided informed assent to participate, with parental written informed consent. From age 18 years, participants provided written informed consent to continue the study as adults.

### 2.1 Study Design and Physical Activity Context

The original repeated measures design aimed to study gymnastics as an extreme model of mechanical loading during youth by comparing serial measures of bone accrual in “non‐gymnasts” versus “gymnasts.” Participation in physical activity was recorded as hours per week (h/week) for each sport reported during semi‐annual measurement sessions up to age 18 years (annually thereafter). “Gymnasts” were defined based on at least 12 months with annual mean training level ≥ 6 h/week; not all gymnasts were competitive, few were “elite,” and exposure was often transient (Dowthwaite et al. 2015). Non‐gymnasts and gymnasts participated in various organized activities by type, intensity, duration, and timing relative to menarche, most commonly gymnastics, dance/aerobics, soccer, track, long‐distance running, basketball, lacrosse, softball/baseball, and volleyball, as well as aquatic, non‐weight‐bearing activities (swimming, diving) (Dowthwaite et al. 2015). Thus, we amassed a longitudinal dataset that is relevant to bone health research and practice in the context of growth and aging, physical activity, and nutrition science.

### 2.2 Variables Relevant to Maturation and Growth

We documented participant ethnic and racialized classifications to meet US National Institutes of Health (NIH) reporting requirements (Lewis et al. 2023). For baseline pubertal status, Tanner breast stage was self‐reported (with parental assistance as needed). At annual DXA sessions, we measured weight (kg), height (m), and calculated body mass index (BMI: weight/height^2^, kg/m^2^). Menarche status was queried at semi‐annual measurement sessions until menarche date was recorded for gynecological age calculation (gynAGE: scan vs. menarche date difference, rounded to 0.1 year). Menarche is a clear maturational landmark, ascertainable by self‐report and indicative of estrogen exposure, central to female BMC accrual. Thus, we use gynAGE as our biological age standard for comparison with chronological age (cAGE).

### 2.3 DXA Methodology

**FIGURE 1:**
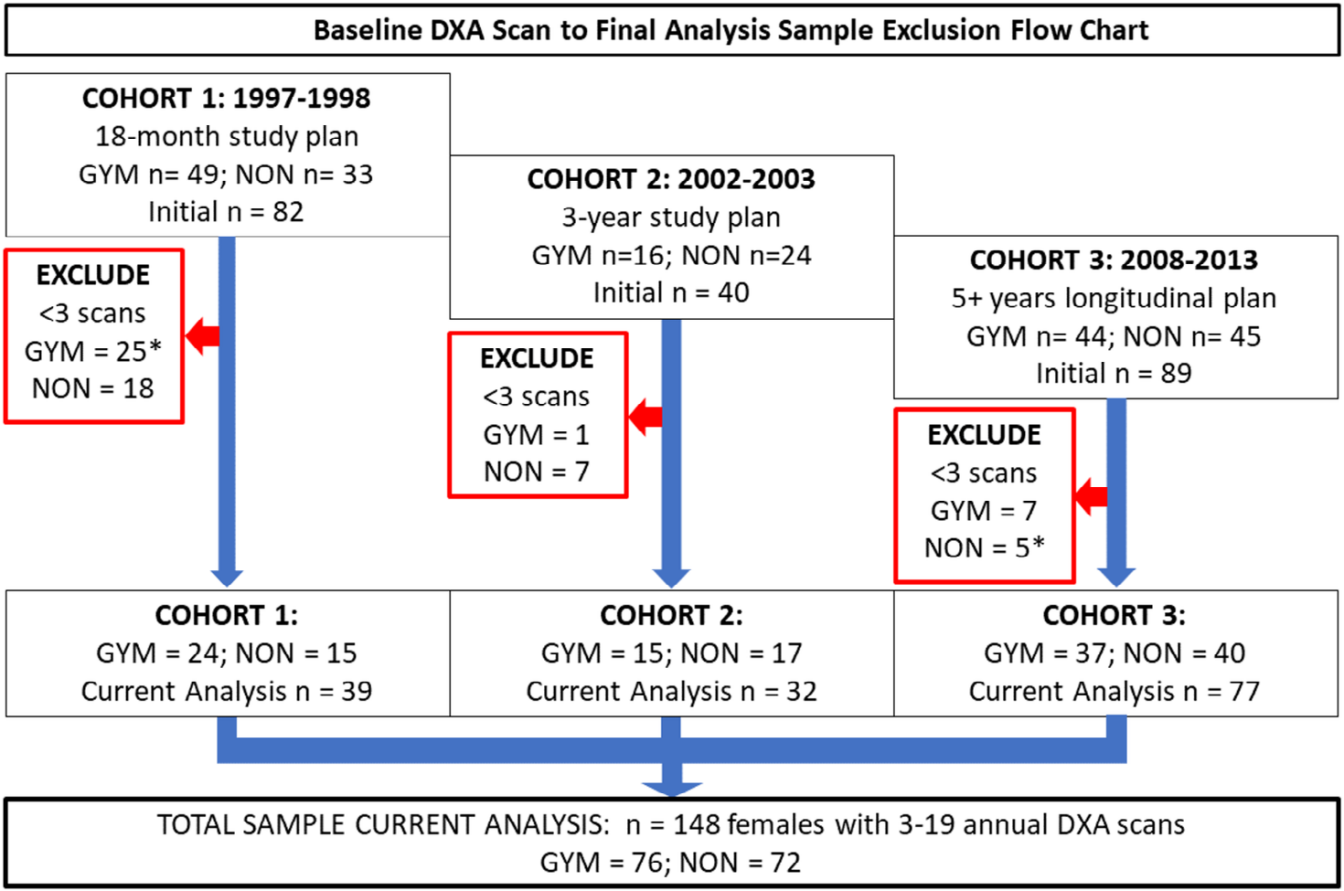
Baseline DXA scan to final analysis sample inclusion flow chart displays breakdown of original longitudinal study participants, exclusion criteria, and breakdown of sample for the current analysis.

Healthy girls were recruited in three cohorts to yield an adequate longitudinal sample for analyses of exercise‐related bone accrual using annual DXA (cAGE 7–15 years at baseline scan, target inter‐scan interval = 0.85 to 1.15 years). For the current work, we included participants who provided at least 3 years of serial whole‐body DXA scans (*n* = 148, Figure 1). Annual WB scans were performed using one of two cross‐calibrated DXA scanners (Hologic, Waltham, MA): QDR4500W (QDR: 1998–2012) or Discovery A (DISCO: 2008–2017). To convert QDR output to DISCO‐equivalent data, we performed contemporaneous duplicate scans in these cohorts to span the focal maturity and body size range (QDR vs. DISCO: *n* = 133, cAGE: 8–24 years) (Dowthwaite et al. 2018). We applied this correction factor to optimize data quality for 32 girls from Cohorts 1 and 2 affected by the QDR‐DISCO scanner change (*n* = 116, no QDR‐DISCO transition). Few scans were affected by this one‐time QDR to DISCO transition (*n* = 32; 1–7 serial scans), so variability within subjects is not due to repeated scanner switching.

Across time series, > 90% of scans were performed by 2 techs (CR, EB), analyzed by one investigator (JD). Regular quality control scans ensured data integrity. Individuals were positioned with heads near the top of the table, wearing light clothing, and removing dense materials whenever possible (lab checks confirmed negligible foreign object influence for smoothed models in the current study). Resultant lab root mean square error coefficients of variation (rmseCV) are: WBBMC 0.8%; SUBBMC 1.0%, and HEADBMC 1.8% (Dowthwaite et al. 2018). Calculated lab LSC values are: WBBMC = 2.22%, SUBBMC = 2.77%, and HEADBMC = 4.99%. Thus, accepted densitometric protocols and lab quality controls yielded high quality serial bone mass data. For each WB scan, HEAD BMC, SUBBMC, and WBBMC were generated (g). HEAD:WB BMC was calculated as a ratio to directly quantify the time‐varying proportion of WBBMC contributed by the HEAD. Variation of this proportion as a function of cAGE and gynAGE is our focus, as the excess contribution of HEADBMC versus sub‐head BMC to WBBMC during childhood is central to the rationale for excluding HEADBMC from pediatric bone assessments.

### 2.4 Statistical Analysis

We used SPSS v.23 (IBM, Armonk, NY) to compare baseline characteristics for excluded versus included participants (independent sample *t*‐tests, χ^2^). Super‐Imposition by Translation and Rotation (R SITAR package v.3.4, 4 df, optimal BIC) was used to model height data and extract individual cAGEs at peak height velocity (Cole et al. 2010), in a sub‐sample classified as White and non‐Hispanic. We used SAS/STAT 9.4 software (SAS Institute, Cary, NC) for all other analyses. We calculated baseline descriptive statistics (sample size, means, standard deviations, 95% confidence intervals (95% CI)) to summarize study participant characteristics by cAGE (even years, 8 to 28) and gynAGE (even years, −6 to +14) strata. We summarize HEAD:WB BMC ratios for key cAGE ranges (cAGE: ≤ 10 years, ≥ 18 years) and maturity phases (pre‐menarche, circum‐menarche, adult, if available; circum‐menarche, closest scan to gynAGE = 0, −1 to +1 year); data are provided as “means of means” to yield an overall arithmetic mean (qualifying observations were averaged per‐individual; per‐individual means were averaged). Finally, we used cubic smoothing spline mixed effects models, with 95% CIs, to generate cAGE and gynAGE growth curves for DXA output (WBBMC, HEADBMC, SUBBMC, HEAD:WB BMC). The current sample size was not determined a priori for the specific purpose of testing the hypothesis addressed in the current paper. Instead, the sample size reflects available data collected to evaluate different hypotheses related to maturity‐specific bone accrual. Gymnasts and non‐gymnasts were not subdivided for comparison in the current analysis, as we aimed to summarize growth patterns across heterogeneous loading exposures.

## 3 Results

### 3.1 Descriptive Statistics

Our cAGE analysis included 1172 scans, representing 148 females (gymnast *n* = 76; non‐gymnast *n* = 72); 63 girls were excluded due to < 3 annual scans (gymnast *n* = 33, non‐gymnast *n* = 30). For the baseline scan, most indicated pre‐pubertal or early pubertal Tanner breast (TB) stages: TB1 (*n* = 73), TB2 (*n* = 64), (TB3, *n* = 11). Most included participants identified as White, non‐Hispanic (*n* = 135: 91.9% of sample); other reported identities included: 7 Asian (4.7%), 4 multiracial (2 Black/White, 2 Asian/White: 2.7%), and 1 White, Hispanic (0.7%). We did not detect differences for included vs. excluded individuals for baseline age, height, weight, BMI, WBBMC, lean mass, % body fat, or non‐aquatic physical activity (*t*‐test, *p* > 0.20); racialized or ethnic identity, gymnast status (*χ* ^2^, *p* > 0.65) or Tanner breast stage (*χ* ^2^, *p* = 0.29).

General and baseline characteristics are presented for included participants (Table 1). Repeated DXA scans ranged from 3 to 19 assessments per girl (mean 8, sd 3.3), yielding broad cAGE ranges (baseline: 7.6–15.1 years, “latest scan”: 10.5–30.0 years). Across the dataset, the mean interscan interval was 1.15 years (sd 0.47), accounting for missing data and irregularities in annual scan timing. Scan numbers by cAGE and gynAGE are presented in Supporting Information (Tables S1 and S2). In the cAGE analysis, 13 girls provided only 3 annual scans (< 3%). Due to missing menarche dates, the gynAGE analysis sample was slightly smaller (*n* = 132): all 132 provided ≥ 4 years of scans, and 97.7% provided scans pre‐ and post‐menarche. Most individuals excluded from the gynAGE analysis were not observed post‐menarche due to discontinuing study participation before menarche (mainly Cohort 1) or not achieving menarche by the time of analysis (Cohort 3); one excluded participant did not achieve spontaneous menarche and was prescribed hormonal contraceptive therapy. Seven participants reported menarcheal age ≥ 15 years (5.3%), while three additional participants had still not achieved menarche by age 15 years at their most recent scan. Fifteen girls provided only pre‐menarcheal scans (10.1%), while 3 provided only post‐menarcheal scans (2%: initial scan gynAGE +0.4 years, +0.6 years, +3.0 years). Core cAGE (10 to 14 years) and gynAGE (−2 to +2 years) ranges were represented by ≥ 75 individuals throughout. A large subset of the current sample (*n* = 141) demonstrated mean cAGE at peak height velocity of 12.3 years (extracted from SITAR models); thus, assessments beyond cAGE 12 years likely reflect post‐PHV status. The mean for self‐reported weight‐bearing physical activity over the 12 months leading to menarche was 8.7 h/week (annual mean: sd 5.6; min 0, max 22.6, 95% CI 7.8–9.7).

**TABLE 1:** Descriptive Statistics of study sample for age characteristics and baseline traits.

Variable	*n*	Mean (sd)	Min–max
Mean number of scans	148	7.9 (3.3)	3, 19
Baseline chronological age (Age, years)	148	10.3 (1.5)	7.6, 15.1
Latest scan chronological age (Age, years)	148	18.2 (4.2)	10.5, 30.0
Age at peak height velocity (years)	141	12.27 (0.87)	10.17, 14.98
Age at menarche (years)	132	13.06 (1.24)	9.55, 17.41
Baseline gynecological age (years)	132	−2.7 (1.8)	−7.9, +3.0
Latest scan gynecological age (years)	132	+5.7 (4.0)	−1.6, +17.0
Baseline weight (kg)	148	34.16 (9.12)	19.80, 72.60
Baseline height (cm)	148	138.30 (10.38)	116.00, 166.70
Baseline BMI (kg/m^2^)	148	17.58 (2.65)	13.54, 27.96
Baseline WB BMC (g)	148	1188.44 (278.50)	808.39, 2595.45
Baseline head BMC (g)	148	287.49 (39.58)	184.02, 425.23
Baseline SUB BMC (g)	148	901.49 (251.97)	556.95, 2170.22
Baseline HEAD:WB BMC	148	0.25 (0.04)	0.16, 0.34

Abbreviations: BMC = bone mineral content; BMI = body mass index; sd = standard deviation; SUB BMC = sub‐cranial BMC (total body, less head); WB BMC = whole body bone mineral content.

### 3.2 Adult Head to Whole‐Body Proportions

**FIGURE 2:**
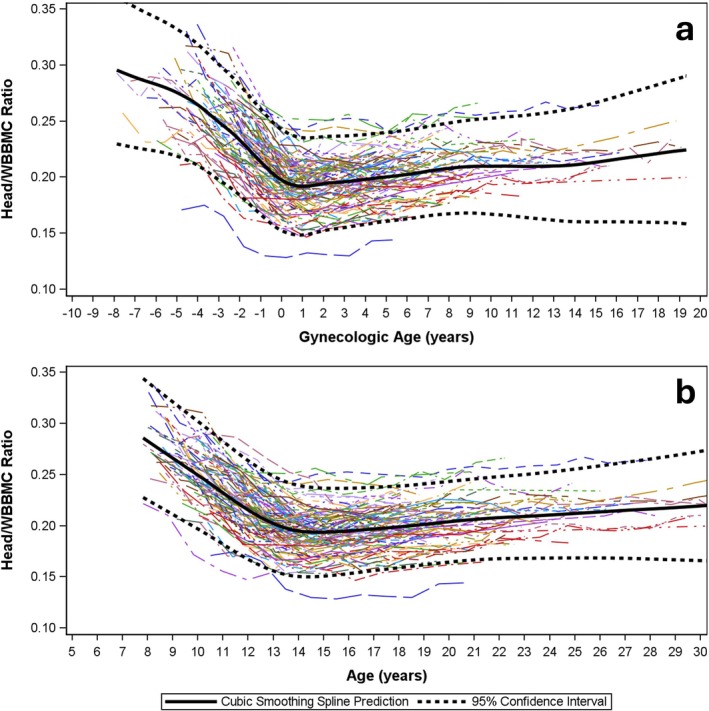
Modeled data represent HEAD:WB BMC change from childhood to adulthood using *gynecological* (*2a*) and *chronological* age‐based curves (*2b*). Sample means are bold, black solid curves; 95% confidence intervals for mean curves are bold, black, dashed curves. Individual growth curves are represented using unique combinations of curve colors and patterns.

**FIGURE 3:**
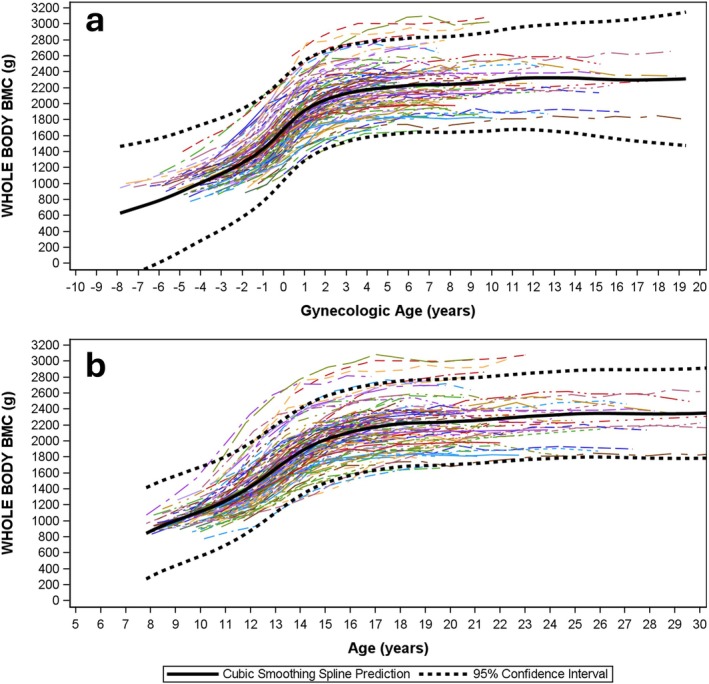
Modeled data represent WBBMC change from childhood to adulthood using *gynecological* (*3a*) and *chronological* age‐based curves (*3b*). Sample means are bold, black solid curves; 95% confidence intervals for mean curves are bold, black, dashed curves. Individual growth curves are represented using unique combinations of curve colors and patterns.

**FIGURE 4:**
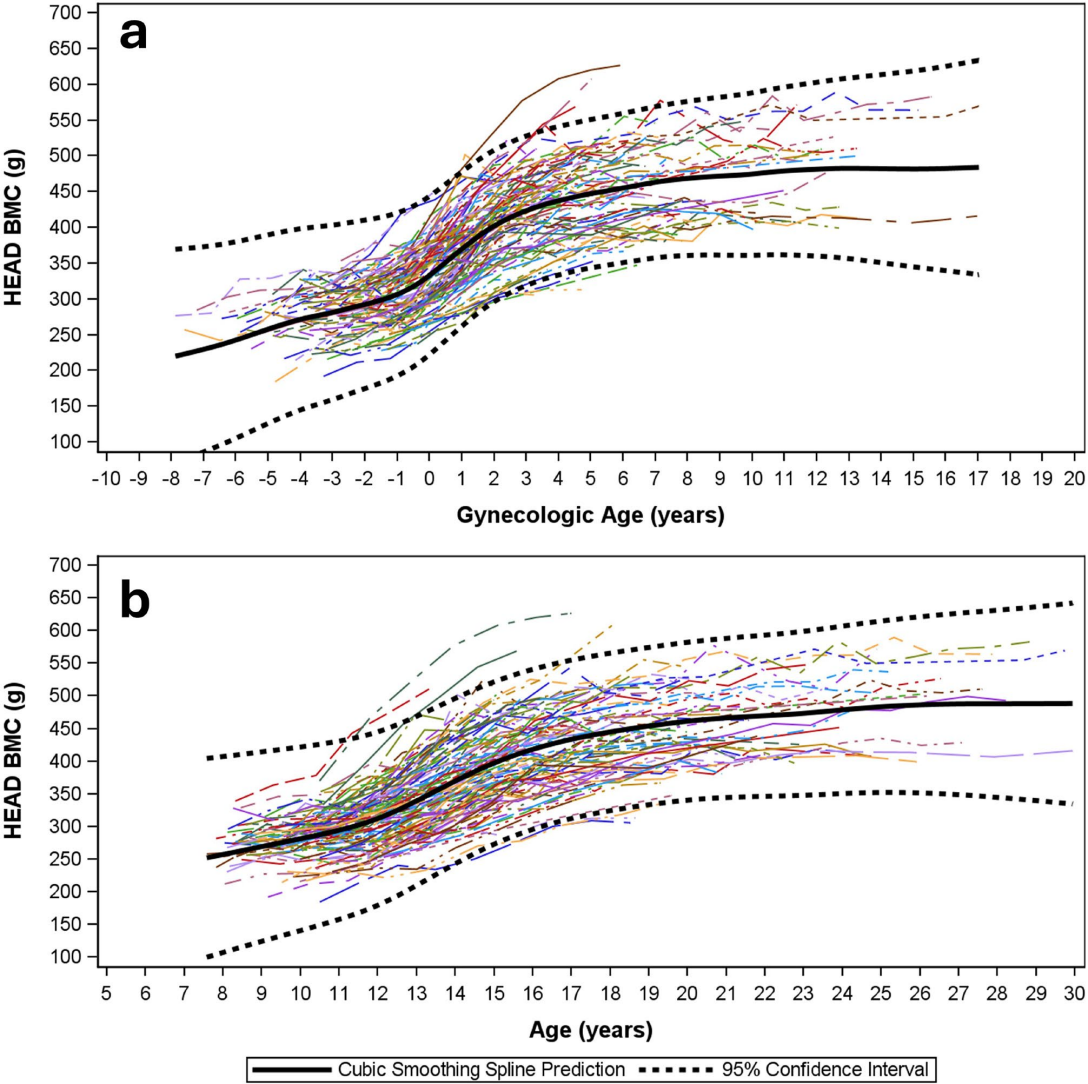
Modeled data represent HEADBMC change from childhood to adulthood using *gynecological* (*4a*) and *chronological* age‐based curves (*4b*). Sample means are bold, black solid curves; 95% confidence intervals for mean curves are bold, black, dashed curves. Individual growth curves are represented using unique combinations of curve colors and patterns.

**FIGURE 5:**
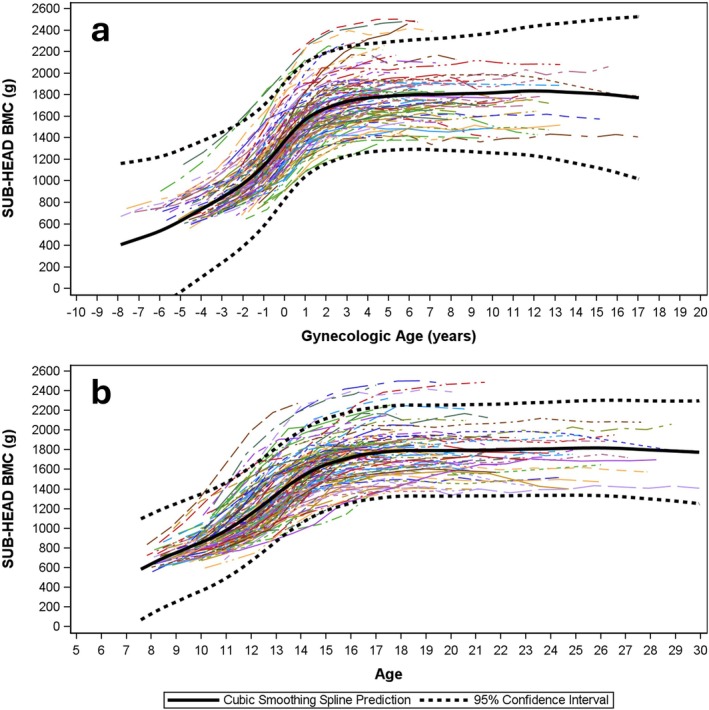
Modeled data represent SUBBMC change from childhood to adulthood using *gynecological* (*2a*) and *chronological* age‐based curves (*2b*). Sample means are bold, black solid curves; 95% confidence intervals for mean curves are bold, black, dashed curves. Individual growth curves are represented using unique combinations of curve colors and patterns.

Modeled data for HEAD:WB BMC are presented by gynAGE (Figure 2a) and cAGE curves (Figure 2b). Comparable curves are presented for WBBMC (Figure 3a,b), HEADBMC (Figure 4a,b) and SUBBMC (Figure 5a,b). Childhood HEAD:WB BMC means (age 8–10 years) were high: mean for ages ≤ 10 years = 0.26 (0.03) (Table 2). Minimum interpolated means for HEAD:WB BMC were roughly 0.20 (0.02) at cAGE 14.0 years, gynAGE 0 years, and at the closest measurement to menarche (circum‐menarche, Table 2). HEAD:WB BMC stabilized at roughly 0.20 to 0.21 (0.02) across all post‐menarcheal data, and 95% CI for “adult” proportions overlapped from cAGE 12.0 years onwards, with no noteworthy difference from age 12 years or early post‐menarche to adult. Individual and mean growth curves, presented by gynAGE and cAGE (Figure 2a,b), support use of these thresholds for attainment of adult HEAD:WB proportions. Inspection of gynAGE‐based results for girls classified to represent non‐white and Hispanic identities showed that curves fit within total sample 95% CI. Figure 4a,b show high variability in HEADBMC across time, particularly for later gynAGE and cAGE, indicating spurious variation attributable to differences in head position and/or scan sub‐division.

**TABLE 2:** Descriptive statistics for HEAD:WB BMC proportion for different maturity and chronological age phases.

Time point	*n*	Mean (sd)	Min–max	95% CI
Pre‐menarche	129	0.227 (0.03)	0.16, 0.30	0.223, 0.232
Circum‐menarche	124	0.198 (0.02)	0.13, 0.25	0.194, 0.202
Post‐menarche	125	0.198 (0.02)	0.13, 0.25	0.194, 0.202
Prior to age 10 years	65	0.265 (0.03)	0.19, 0.32	0.257, 0.271
After age 18 years	66	0.204 (0.02)	0.13, 0.26	0.198, 0.210

*Note:* Circum‐menarche = closest observation to menarche within gynecological age range −1.0 to +1.0 years.

Abbreviations: BMC = bone mineral content; sd = standard deviation; WB = whole‐body.

### 3.3 Whole‐Body Proportions

Comparisons of curves for WBBMC and SUBBMC (Figure 3 vs. Figure 4) show the effect of removing HEADBMC; these curves have similar growth patterns with clear plateaus, indicating attainment of peak bone mass. Smoothed black SUBBMC mean curves minimize the influence of measurement error added by subdividing scans to remove HEADBMC, whereas intra‐individual HEADBMC fluctuations are highlighted (Figure 4), partly due to the larger proportion of error versus measurement (mean adult SUBBMC and WBBMC are 3–4× greater than HEADBMC). Note that HEADBMC growth continues a subtle upward trajectory, suggesting peak bone mass has not been attained. This lack of HEADBMC plateau in early adulthood contributes to a faint rebound in HEAD:WB BMC, with a subdued increasing trend after menarche and cAGE 14 years. In contrast to broad, flattened cAGE curves, gynAGE curves narrow 95% CI for the 4 years of maximum growth velocity centered at menarche (gynAGE: −2 to +2 years), thereby sharply delineating the sigmoid curve of adolescent growth.

## 4 Discussion

Our longitudinal results support our hypothesis: for healthy US girls, including youths with diverse physical activity exposures, mean HEAD:WB BMC proportions are ≤ 21%, analogous to adult proportions, from menarche or age at peak height velocity onwards (mean PHV cAGE = 12.3 years). Accordingly, beyond these benchmarks, WBBMC may be used to assess bone mass adequacy for cAGE and height Z‐score, simplifying bone accrual evaluation in adolescent girls. The use of WBBMC rather than SUBBMC should decrease measurement error and reduce LSC to optimize research monitoring and clinical decisions during the critical period from menarche to peak bone mass. We do not recommend SUBBMC monitoring for individuals who are post‐menarcheal at baseline. SUBBMC monitoring is only relevant prior to attainment of adult proportions (pre‐menarche). For clinical cases or longitudinal studies that span childhood to post‐menarche, after menarche, we recommend plotting the full WBBMC trajectory for age and height Z‐score, ideally centered at menarche, to avoid a sudden step change from assessing SUBBMC to assessing WBBMC in later analyses.

Other studies support our contention that exclusion of HEAD BMC increases measurement error for adolescent bone assessments. Over the key adolescent transition to peak bone mass (age 13–24 years), use of SUBBMC instead of WBBMC decreases reliability, boosting error and inflating LSC by 60% (SUBBMC: rmseCV = 4.8%, LSC = 13.3%; WBBMC: rmseCV = 3.0%, LSC = 8.3%) (Dowthwaite et al. 2018). Accordingly, for age 13–17 years, a smaller change in WBBMC would meet LSC criteria to trigger treatment decisions at a lower threshold than would be required for standard pediatric SUBBMC monitoring. Others have shown that erroneously low or high BMC assessments are likely in late adolescence after BMC accrual rates have plateaued (Baxter‐Jones et al. 2011), with short‐term fluctuations suggesting “negative change” in BMC commonly observed in healthy individuals. In our current analysis, post‐menarcheal fluctuations are not attributable to the QDR‐DISCO scanner change; they likely reflect differences in head and reference line positioning, not bone mass changes, because fluctuations are lower in WBBMC than SUBBMC curves (Figure 4 vs. Figure 5) and highest for HEAD:WB BMC (Figure 2). As head positioning and reference line placement and other sources of post‐menarcheal HEADBMC variation are irrelevant to fracture risk and apply equally to teens and young adults, SUBBMC monitoring in post‐menarcheal girls may confound research and clinical interpretation in youth athletes, especially those with suspected REDs.

Individuals who exhibit extreme maturational delay should be evaluated with caution, but as long as HEAD:WB BMC proportions are 21% or lower, they are within the mean range for adults. An observed HEAD:WB BMC nadir and/or trough at ≤ 25% beyond PHV or age 12 years also suggests that adult proportions have been reached in girls with higher than average adult HEAD:WB BMC proportions. Of note, our sample included many girls with late menarche. Thus, our analysis accounts for possible delayed maturation seen in the general population due to heritable traits; late menarche is especially common in athletes and others with low energy availability. To be conservative, for late maturers with primary amenorrhea, annual height velocities should be assessed to screen for linear growth deficits and HEAD:WB BMC proportions should be monitored; if warranted, pediatric SUBBMC assessments should continue until menarche is documented. Overall, for adolescent practice, a shift from monitoring SUBBMC to monitoring WBBMC is justifiable after exceeding one or more of the following benchmarks: menarche, cAGE 12 years, demonstrated age at PHV, HEAD:WB BMC trough ≤ 25%, and/or attainment of HEAD:WB BMC ratio ≤ 21%.

Other work has compared the HEAD as a “non‐loaded” site vs. post‐cranial “loaded sites” in the context of youth sports. In a prepubertal comparison of gymnasts, swimmers, and non‐athletes, Courteix et al. reported lower HEADBMC, HEADBMD, and HEAD:WB BMC in gymnasts, interpreting differences as a compensatory BMC drain from gymnasts' non‐loaded skulls to reinforce loaded sites against extreme weight‐bearing impacts (Courteix et al. 1999). Yet, with more careful accounting for height, weight, skeletal age, and prepubertal serum estradiol levels, Bass et al. found no differences between pre‐pubertal elite gymnasts and non‐gymnasts for HEADBMD Z‐scores or 12‐month BMC gains; they also detected no HEADBMD differences for height‐ and weight‐matched adult ex‐gymnasts vs. non‐gymnasts (Bass et al. 1998). In prior menarche‐centered longitudinal analyses, we compared non‐gymnasts versus ex‐gymnasts who quit circum‐menarche, accounting for time‐varying height, non‐bone fat‐free mass, and calcium intake to detect persistent loading‐related advantages in radius DXA properties for ex/gymnasts (Scerpella et al. 2011); we detected no group differences for HEADBMC or HEADBMD (longitudinal *p* > 0.8) and found no evidence of gymnast “BMC drain” from the head to bolster loaded sites (Scerpella et al. 2011). Thus, overall, osteogenic exercise likely improves bone accrual at post‐cranial sites without draining HEADBMC. Overall, by representing menarche‐centered patterns of HEADBMC, SUBBMC, WBBMC, and HEAD:WB BMC for individuals with diverse patterns of youth mechanical loading exposure and withdrawal, our current analyses present a broad range of bone accrual patterns for US middle‐class females in an appropriate maturational context. Our current analysis includes youth athletes representing many types, intensities, and timings of exercise, and bone‐loading exposure. In this context, HEADBMC lacks a clear early adult plateau, suggesting no pervasive decrease in HEADBMC in late adolescence or early adulthood, after discontinuation of scholastic/intercollegiate sports. We are confident that by including gymnasts and non‐gymnasts to evaluate heterogeneous youth exercise exposures, we are summarizing overarching bone accrual patterns relevant to endocrinology, nutrition, exercise science, and sports medicine research and practice in the United States.

A life course approach posits that exposures to potent environmental factors during critical and sensitive periods of development may have synergistic influences on physical maturation, somatic growth, bone accrual patterns, and overall susceptibility to chronic conditions such as osteoporosis (Mishra et al. 2010). Early life influences on maturation rates have been demonstrated by recent analyses from the Avon Longitudinal Study of Parents and Children (ALSPAC: 1991–1992 UK birth cohort). ALSPAC data linked higher prepubertal intakes of total calories and protein with earlier pubertal onset, PHV, and menarche in UK females, independent of adolescent DXA % body fat, even after adjusting for key maternal, prenatal, and infant variables to account for early life socioeconomic, environmental, and anthropometric factors (Cheng et al. 2022). A life course perspective was also adopted for growth analyses that combined ALSPAC data with 1940's UK growth data from the National Study of Health and Development (NSHD), to borrow strength for extraction of the SITAR pubertal timing (TEMPO) metric (Cole et al. 2016). In the NSHD sample, earlier timing of pubertal growth for height and weight was positively correlated with radius metaphysis trabecular vBMD and diaphysis cross‐sectional area at age 60–64 years (Cole et al. 2016). Like our findings, these life course data indicate potential sensitive periods in early life, and demonstrate the value of biological benchmarks, such as ages at menarche and PHV for analyses of nutrition, exercise, and other factors in the development of body composition, bone properties, and chronic disease risk.

### 4.1 Limitations

Longitudinal data analyses provided challenges via loss‐to‐follow‐up and equipment changes. Data after cAGE 18 years and gynAGE +6 years represent fewer individuals, resulting in broader 95% CI. In the higher age ranges, it is unclear whether subtle patterns of increasing HEAD:WB BMC apply to all individuals or are due to a limited mature sample. Nonetheless, adult trajectories appear consistent and stable, as expected after cessation of linear growth. Our analyses excluded data for ~30% of main longitudinal study participants who provided few annual observations; we detected no attrition/exclusion bias in baseline descriptive statistics. With > 97% of eligible scans included for the relevant age range (674/690) and > 1100 scans overall, we are confident that our main findings have not been compromised. We are also confident that our DXA quality control practices support valid conclusions, as observed HEAD:WB BMC growth trajectories were consistent across > 130 individuals, with negligible potential for scanner change confounding. Overall, we provide a wealth of high‐quality data representing key growth periods from pre‐puberty into early adulthood.

As females are underrepresented in the literature and are more prone to fragility fractures, our data address a vital population of concern. Because our sample is wholly female, our data do not apply to males. Males have longer growth periods with later peak velocities for height and BMC accrual; based on 97% peak bone mass attainment (Baxter‐Jones et al. 2011), we expect male HEAD:WB BMC proportions to reach a nadir 2–3 years later than females. Further work is needed to test this hypothesis in males, particularly males at risk of REDs. Our participants were healthy at baseline and remained broadly healthy; thus, our results may not generalize to special populations or individuals affected by congenital bone pathologies or other chronic diseases. As our participants were originally recruited as gymnasts or non‐gymnasts, and engaged in a broad variety of sports, our data reflect normally active participants with heterogeneous and variable loading exposures and energetic challenges across growth; we view this design as a strength for the application of our findings to active populations.

Our sample was fairly homogeneous (> 90% White, non‐Hispanic identity). Socioeconomic status was not assessed, but extended longitudinal study participation suggests high access to socioeconomic resources (transportation, education, nutrition, medical services). Other research groups have reported differences attributed to racialized and/or ethnic identities for bone maturation (Zhang et al. 2009), bone mineral accrual (Burrows, Baxter‐Jones, Mirwald, MacDonald, McKay, Burrows et al. 2009), and bone microarchitecture (Misra et al. 2017); thus, HEAD:WB BMC patterns may vary accordingly, likely as a function of maturational differences. For example, viewed through the lens of NIH reporting criteria, US girls categorized as Black or Hispanic may achieve menarche earlier than girls who identify as White or Asian, possibly due to socioeconomic factors specific to populations studied in the United States (Deardorff et al. 2014). These observed patterns lend support to life course perspectives on environmental influences such as socioeconomic factors in female development and health (Mishra et al. 2010). However, genomic factors may also be involved, as Mendelian Randomization analyses linked polygenic risk factors to earlier pubertal onset, age at menarche, adolescent lumbar spine BMD, and adult BMD at lumbar spine and femoral neck in a large multi‐site sample limited to US females of European ancestry (Cousminer et al. 2018). The International Society for Clinical Densitometry recently questioned the practice of requesting racialized or ethnic identity from US pediatric densitometry patients (Ramadan et al. 2025). Our data support the view that these categories may be less relevant in bone research and clinical practice if biological benchmarks are substituted to account for interindividual variability, emphasizing menarche, PHV, and HEAD:WB BMC ≤ 21% rather than cAGE thresholds. It is unclear whether comparable biological benchmarks hold across populations; biological age‐based curves should be generated to test this premise by collecting longitudinal data from more diverse US samples and samples drawn from non‐US geographical and socioeconomic populations. Additional research is needed to elucidate the relative influence of the environment, genome, transcriptome, and microbiome on physical maturation and bone accrual in varied contexts.

## 5 Conclusion

US girls attain adult HEAD:WB proportions by menarche. Thus, after menarche, WBBMC may be used to monitor bone accrual to minimize measurement error and reduce LSC, even in late maturers. This practice may optimize bone accrual assessment during this critical maturational period leading to peak bone mass. If the menarche date is unknown, but maturation appears early or normal, HEAD:WB BMC ≤ 21% (or observed nadir/trough ≤ 25%), post‐PHV status, and/or age > 12 years may indicate appropriate thresholds for transition from SUBBMC to WBBMC monitoring in the approach to peak bone mass. Soon, we will perform extended longitudinal analyses to evaluate youth exercise and nutrition as maturity‐specific factors in BMC accrual patterns from childhood into early adulthood. Future research should determine patterns in males and individuals of diverse racial, ethnic, socioeconomic, and medical characteristics and test for long‐term stability of adult BMC proportions into perimenopause and senescence.

## Disclosure

The results of the study are presented clearly, honestly, and without fabrication, falsification, or inappropriate data manipulation.

## Ethics Statement

Our human subjects research study protocols have been approved by the Institutional Review Board of SUNY Upstate Medical University and comply with the Declaration of Helsinki.

## Conflicts of Interest

The authors declare no conflicts of interest.

## Supporting information


**Table S1:** Descriptive statistics by chronological age strata.

**Table S2:** Descriptive statistics by gynecological age strata.

## Data Availability

The data that supports the findings of this study are available in the Supporting Information of this article.
