# Supplementary material for: Biological Benchmarks for Adult Bone Mass Proportions in Young Females: A Prospective Longitudinal Analysis
Source: Am J Hum Biol. 2025 Aug 13;37(8):e70118. doi: 10.1002/ajhb.70118 (PMC12344745; doi:10.1002/ajhb.70118)
Supplement: Supplementary file 1 — Table S1: Descriptive statistics by chronological age strata. [file AJHB-37-e70118-s002.docx]

| **Table S1:** Descriptive Statistics by Chronological Age Strata | | | | | |
| --- | --- | --- | --- | --- | --- |
| **Age** | **Variable** | **n** | **Mean (sd)** | **Min, Max** | **95% CI** |
| **8** | **Gynecological Age (yrs)** | 27 | -4.15 (1.55) | -7.87, -0.66 | -3.54, -4.77 |
|  | **Weight (kg)** | 35 | 27.50 (5.50) | 19.80, 50.20 | 25.61, 29.39 |
|  | **Height (cm)** | 35 | 128.39 (6.66) | 116.00, 143.00 | 126.10, 130.38 |
|  | **BMI (kg/m^2^)** | 35 | 16.58 (2.07) | 13.54, 24.62 | 15.86, 17.29 |
|  | **WB BMC (g)** | 35 | 991.40 (109.34) | 831.55, 1282.60 | 953.84, 1028.96 |
|  | **Head BMC (g)** | 35 | 275.26 (26.58) | 211.64, 338.43 | 266.13, 284.40 |
|  | **SUB BMC (g)** | 35 | 715.93 (101.56) | 556.95, 1013.11 | 681.04, 750.82 |
|  | **Head:WB BMC** | 35 | 0.28 (0.03) | 0.20, 0.34 | 0.27, 0.29 |
| **10** | **Gynecological Age (yrs)** | 93 | -2.52 (1.39) | -6.48, 0.40 | -2.81, -2.24 |
|  | **Weight (kg)** | 107 | 34.70 (6.95) | 21.30, 68.40 | 33.37, 36.03 |
|  | **Height (cm)** | 107 | 139.73 (6.62) | 123.00, 159.00 | 138.46, 141.00 |
|  | **BMI (kg/m^2^)** | 107 | 17.64 (2.37) | 14.08, 27.06 | 17.18, 18.09 |
|  | **WB BMC (g)** | 107 | 1193.39 (169.06) | 808.69, 1953.18 | 1160.99, 1225.80 |
|  | **Head BMC (g)** | 107 | 288.33 (35.90) | 184.02, 377.80 | 281.45, 295.21 |
|  | **SUB BMC (g)** | 107 | 905.69 (151.65) | 594.45, 1649.36 | 876.62, 934.75 |
|  | **Head:WB BMC** | 107 | 0.24 (0.03) | 0.16, 0.31 | 0.24, 0.25 |
| **12** | **Gynecological Age (yrs)** | 112 | -0.53 (1.30) | -4.62, 3.33 | -0.77, -0.29 |
|  | **Weight (kg)** | 120 | 44.25 (8.51) | 25.90, 73.60 | 42.71, 45.79 |
|  | **Height (cm)** | 120 | 152.47 (7.38) | 132.50, 170.20 | 151.13, 153.80 |
|  | **BMI (kg/m^2^)** | 120 | 18.91 (2.54) | 14.75, 27.96 | 18.45, 19.37 |
|  | **WB BMC (g)** | 120 | 1560.96 (286.62) | 968.81, 2572.83 | 1509.15, 1612.77 |
|  | **Head BMC (g)** | 120 | 326.38 (50.34) | 221.08, 524.99 | 317.28, 335.48 |
|  | **SUB BMC (g)** | 120 | 1236.29 (249.84) | 752.29, 2177.77 | 1191.13, 1281.45 |
|  | **Head:WB BMC** | 120 | 0.211 (0.02) | 0.15-0.26 | 0.207,0.216 |
| **14** | **Gynecological Age (yrs)** | 105 | 1.28 (1.25) | -2.59, 5.28 | 1.04,1.52 |
|  | **Weight (kg)** | 113 | 53.06 (8.17) | 35.00, 83.00 | 51.53, 54.58 |
|  | **Height (cm)** | 112 | 159.59 (6.88) | 142.90, 179.90 | 158.30, 160.88 |
|  | **BMI (kg/m^2^)** | 112 | 20.80 (2.70) | 15.59, 30.93 | 20.29, 21.31 |
|  | **WB BMC (g)** | 113 | 1953.61 (279.51) | 1344.65, 2799.77 | 1901.51, 2005.71 |
|  | **Head BMC (g)** | 113 | 379.21 (57.16) | 249.80, 543.56 | 368.55, 389.86 |
|  | **SUB BMC (g)** | 113 | 1576.65 (243.95) | 1041.64, 2330.77 | 1531.18, 1622.13 |
|  | **Head:WB BMC** | 113 | 0.195 (0.02) | 0.13, 0.26 | 0.191, 0.199 |
| **16** | **Gynecological Age (yrs)** | 85 | 3.28 (1.27) | -0.61, 7.33 | 3.00, 3.55 |
|  | **Weight (kg)** | 87 | 58.36 (9.36) | 39.80, 94.40 | 56.37-60.36 |
|  | **Height (cm)** | 87 | 162.47 (6.66) | 145.60, 177.00 | 161.05, 163.89 |
|  | **BMI (kg/m^2^)** | 87 | 88.09 (2.99) | 17.69, 31.95 | 21.45, 22.72 |
|  | **WB BMC (g)** | 87 | 2165.98 (277.17) | 1620.84, 3004.79 | 2106.90, 2225.05 |
|  | **Head BMC (g)** | 87 | 421.43 (57.10) | 299.60, 619.75 | 409.26, 433.60 |
|  | **SUB BMC (g)** | 87 | 1746.81 (246.35) | 1327.31, 2462.19 | 1694.30, 1799.31 |
|  | **Head:WB BMC** | 87 | 0.20 (0.02) | 0.13, 0.24 | 0.191, 0.200 |
| **18** | **Gynecological Age (yrs)** | 46 | 5.36 (0.94) | 3.24, 7.17 | 5.08, 5.64 |
|  | **Weight (kg)** | 47 | 59.29 (9.03) | 43.40, 95.40 | 56.64, 61.94 |
|  | **Height (cm)** | 47 | 162.78 (6.75) | 146.70, 175.00 | 160.79, 164.76 |
|  | **BMI (kg/m^2^)** | 47 | 22.33 (2.72) | 18.03, 31.73 | 21.53, 23.13 |
|  | **WB BMC (g)** | 47 | 2210.65 (262.76) | 1711.57, 3000.06 | 2133.51, 2287.80 |
|  | **Head BMC (g)** | 47 | 442.74 (64.52) | 305.29, 607.23 | 423.79, 461.68 |
|  | **SUB BMC (g)** | 47 | 1768.86 (232.89) | 1401.36, 2500.29 | 1700.48, 1837.24 |
|  | **Head:WB BMC** | 47 | 0.20 (0.02) | 0.13-0.25 | 0.194, 0.208 |
| **20** | **Gynecological Age (yrs)** | 35 | 7.24 (1.04) | 4.73, 9.35 | 6.88, 7.60 |
|  | **Weight (kg)** | 36 | 58.57 (10.86) | 40.60, 109.60 | 54.90, 62.25 |
|  | **Height (cm)** | 36 | 162.66 (7.06) | 146.80, 173.40 | 160.27, 165.05 |
|  | **BMI (kg/m^2^)** | 36 | 22.09 (3.39) | 17.99, 36.45 | 20.95, 23.24 |
|  | **WB BMC (g)** | 36 | 2200.25 (268.51) | 1740.03,2912.51 | 2109.40, 2291.10 |
|  | **Head BMC (g)** | 36 | 458.66 (51.81) | 379.63, 577.21 | 441.13, 476.19 |
|  | **SUB BMC (g)** | 36 | 1741.65 (248.55) | 1335.94, 2387.05 | 1657.55, 1825.75 |
|  | **Head:WB BMC** | 36 | 0.21 (0.02) | 0.16-0.26 | 0.202,0.218 |
| **22** | **Gynecological Age (yrs)** | 16 | 8.87 (1.43) | 5.44, 11.13 | 8.11, 9.64 |
|  | **Weight (kg)** | 16 | 55.08 (5.36) | 42.80, 66.80 | 52.82, 57.93 |
|  | **Height (cm)** | 16 | 162.14 (8.03) | 147.00, 173.00 | 157.86, 166.42 |
|  | **BMI (kg/m^2^)** | 16 | 20.93 (1.54) | 18.29, 23.54 | 20.13, 21.78 |
|  | **WB BMC (g)** | 16 | 2182.66 (240.50) | 1769.59, 2561.98 | 2054.51, 2310.82 |
|  | **Head BMC (g)** | 16 | 467.65 (52.42) | 396.86, 540.11 | 439.72, 495.59 |
|  | **SUB BMC (g)** | 16 | 1715.62 (205.82) | 1360.60, 2083.32 | 1605.95, 1825.30 |
|  | **Head:WB BMC** | 16 | 0.21 (0.02) | 0.19, 0.25 | 0.205, 0.224 |
| **24** | **Gynecological Age (yrs)** | 15 | 10.81 (1.29) | 7.36, 12.28 | 10.09, 11.52 |
|  | **Weight (kg)** | 15 | 58.05 (7.31) | 44.80, 71.60 | 54.00, 62.10 |
|  | **Height (cm)** | 15 | 161.77 (8.06) | 147.80, 172.40 | 157.31, 166.24 |
|  | **BMI (kg/m^2^)** | 15 | 22.24 (3.10) | 19.31, 31.40 | 20.53, 23.96 |
|  | **WB BMC (g)** | 15 | 2241.82 (257.74) | 1805.42, 2618.60 | 2099.09, 2384.55 |
|  | **Head BMC (g)** | 15 | 481.91 (62.37) | 406.01, 571.06 | 448.67, 515.14 |
|  | **SUB BMC (g)** | 15 | 1740.36 (215.02) | 1396.27, 2094.86 | 1625.76, 1854.92 |
|  | **Head:WB BMC** | 15 | 0.22 (0.02) | 0.18, 0.26 | 0.207, 0.227 |
| **26** | **Gynecological Age (yrs)** | 8 | 12.29 (1.36) | 9.43, 13.60 | 11.15,13.42 |
|  | **Weight (kg)** | 8 | 64.45 (6.95) | 53.60, 73.80 | 58.64, 70.26 |
|  | **Height (cm)** | 8 | 166.66 (4.03) | 160.90, 171.60 | 163.30, 170.03 |
|  | **BMI (kg/m^2^)** | 7 | 23.77 (2.92) | 19.86, 26.40 | 21.08, 26.47 |
|  | **WB BMC (g)** | 8 | 2337.12 (194.95) | 2147.25, 2587.97 | 2174.14, 2500.11 |
|  | **Head BMC (g)** | 8 | 510.20 (46.27) | 423.86, 571.72 | 471.51, 548.88 |
|  | **SUB BMC (g)** | 8 | 1826.93 (181.92) | 1595.46, 2083.54 | 1674.83, 1979.02 |
|  | **Head:WB BMC** | 8 | 0.22 (0.02) | 0.19, 0.26 | 0.201, 0.237 |
| **28** | **Gynecological Age (yrs)** | 4 | 14.72 (2.19) | 11.45, 15.95 | 11.24, 18.21 |
|  | **Weight (kg)** | 4 | 54.25 (6.85) | 44.20, 59.60 | 43.35, 65.15 |
|  | **Height (cm)** | 4 | 161.75 (11.27) | 147.50, 171.70 | 143.82, 179.68 |
|  | **BMI (kg/m^2^)** | 3 | 20.08 (0.33) | 19.70, 20.32 | 19.26, 20.90 |
|  | **WB BMC (g)** | 4 | 2255.85 (334.51) | 1839.33, 2640.57 | 1723.57, 2788.13 |
|  | **Head BMC (g)** | 4 | 509.79 (76.20) | 410.34, 582.26 | 388.54, 631.03 |
|  | **SUB BMC (g)** | 4 | 1746.07 (260.39) | 1429.00, 2058.31 | 1331.73, 2160.40 |
|  | **Head:WB BMC** | 4 | 0.23 (0.01) | 0.22, 0.24 | 0.216, 0.236 |
| BMI= body mass index; BMC=bone mineral content; WB BMC= whole body bone mineral content;  SUB BMC = sub-cranial BMC (total body, less head); sd= standard deviation | | | | | |
