# Supplementary material for: Biological Benchmarks for Adult Bone Mass Proportions in Young Females: A Prospective Longitudinal Analysis
Source: Am J Hum Biol. 2025 Aug 13;37(8):e70118. doi: 10.1002/ajhb.70118 (PMC12344745; doi:10.1002/ajhb.70118)
Supplement: Supplementary file 2 — Table S2: Descriptive statistics by gynecological age strata. [file AJHB-37-e70118-s001.docx]

| **Table S2:** Descriptive Statistics by Gynecological Age Strata | | | | | |
| --- | --- | --- | --- | --- | --- |
| **Gynecological Age** | **Variable** | **n** | **Mean (sd)** | **Min, Max** | **95% CI** |
| **-6** | **Chron. Age (yrs)** | 5 | 9.45 (1.32) | 7.83-10.93 | 7.82-11.08 |
|  | **Weight (kg)** | 5 | 27.46 (1.97) | 25.4, 30.0 | 25.01, 29.91 |
|  | **Height (cm)** | 5 | 132.90 (5.58) | 124.40, 138.80 | 125.97, 139.83 |
|  | **BMI (kg/m^2^)** | 5 | 15.54 (0.53) | 14.98, 16.41 | 14.88, 16.21 |
|  | **WB BMC (g)** | 5 | 1027.08 (19.79) | 1005.95, 1045.95 | 1002.51, 1051.65 |
|  | **Head BMC (g)** | 5 | 275.56 (21.94) | 241.81, 302.50 | 248.32, 302.80 |
|  | **SUB BMC (g)** | 5 | 750.33 (31.87) | 722.98, 814.14 | 710.76, 789.90 |
|  | **Head:WB BMC** | 5 | 0.268 (0.02) | 0.231, 0.289 | 0.241,0.296 |
| **-4** | **Chron. Age (yrs)** | 28 | 9.54 (1.11) | 8.01, 12.80 | 9.11, 9.97 |
|  | **Weight (kg)** | 28 | 29.36 (5.43) | 20.40,41.70 | 27.26, 31.47 |
|  | **Height (cm)** | 28 | 132.98 (9.19) | 116.00, 149.20 | 129.42, 136.54 |
|  | **BMI (kg/m^2^)** | 28 | 16.48 (1.67) | 14.08, 19.93 | 15.84, 17.13 |
|  | **WB BMC (g)** | 28 | 1043.93 (144.71) | 808.69, 1452.28 | 987.82, 1100.05 |
|  | **Head BMC (g)** | 28 | 272.55 (31.99) | 184.02, 328.71 | 260.15, 284.95 |
|  | **SUB BMC (g)** | 28 | 771.54 (135.74) | 556.95, 1150.26 | 718.90, 824.17 |
|  | **Head:WB BMC** | 28 | 0.264 (0.04) | 0.17, 0.34 | 0.250, 0.278 |
| **-2** | **Chron. Age (yrs)** | 75 | 10.91 (1.15) | 8.80, 14.82 | 10.65, 11.18 |
|  | **Weight (kg)** | 75 | 35.22 (6.37) | 25.60, 64.80 | 33.75, 36.68 |
|  | **Height (cm)** | 75 | 141.49 (7.84) | 127.00, 160.40 | 139.68, 143.29 |
|  | **BMI (kg/m^2^)** | 75 | 17.51 (2.18) | 14.42, 26.90 | 17.01, 18.01 |
|  | **WBBMC (g)** | 75 | 1219.23 (163.61) | 962.29, 1745.33 | 1181.58, 1256.87 |
|  | **Head BMC (g)** | 75 | 291.51 (33.79) | 211.25, 352.97 | 283.74, 299.28 |
|  | **SUB BMC (g)** | 75 | 928.22 (149.68) | 675.72, 1438.02 | 893.79, 962.66 |
|  | **Head:WB BMC** | 75 | 0.24 (0.03) | 0.165, 0.316 | 0.235, 0.248 |
| **0** | **Chron. Age (yrs)** | 124 | 13.05 (1.24) | 9.43, 17.30 | 12.83, 13.27 |
|  | **Weight (kg)** | 124 | 48.22 (7.77) | 33.90, 74.40 | 46.83, 49.60 |
|  | **Height (cm)** | 124 | 155.68 (7.83) | 135.05, 177.15 | 154.29, 157.07 |
|  | **BMI (kg/m^2^)** | 124 | 19.82 (2.42) | 16.37, 27.96 | 19.39, 20.25 |
|  | **WB BMC (g)** | 124 | 1698.24 (277.33) | 1121.42, 2595.45 | 164894, 1747.53 |
|  | **Head BMC (g)** | 124 | 334.51 (44.01) | 249.48, 430.67 | 326.68, 342.33 |
|  | **SUB BMC (g)** | 124 | 1366.19 (251.09) | 846.32, 2170.22 | 1321.55, 1410.82 |
|  | **Head:WB BMC** | 124 | 0.20 (0.02) | 0.13, 0.25 | 0.194, 0.204 |
| **2** | **Chron. Age (yrs)** | 93 | 15.39 (1.25) | 11.90, 19.21 | 15.14, 15.65 |
|  | **Weight (kg)** | 93 | 57.32 (8.11) | 39.80, 84.40 | 55.65, 58.99 |
|  | **Height (cm)** | 93 | 161.69 (6.57) | 145.50, 178.00 | 160.33, 163.04 |
|  | **BMI (kg/m^2^)** | 93 | 21.89 (2.55) | 17.69, 30.93 | 21.37, 22.42 |
|  | **WB BMC (g)** | 93 | 2108.95 (277.78) | 1497.50, 2905.65 | 2051.74, 2166.16 |
|  | **Head BMC (g)** | 93 | 412.96 (53.56) | 277.88, 576.66 | 401.93, 424.00 |
|  | **SUB BMC (g)** | 93 | 1698.17 (250.96) | 1186.26, 2415.58 | 1646.49, 1749.85 |
|  | **Head:WB BMC** | 93 | 0.20 (0.02) | 0.13, 0.25 | 0.192, 0.202 |
| **4** | **Chron. Age (yrs)** | 62 | 17.29 (1.36) | 13.87, 21.14 | 16.94, 17.63 |
|  | **Weight (kg)** | 62 | 60.29 (10.34) | 41.40, 95.40 | 57.66, 62.92 |
|  | **Height (cm)** | 62 | 162.51 (7.40) | 146.0, 180.0 | 160.63, 164.39 |
|  | **BMI (kg/m^2^)** | 62 | 22.79 (3.23) | 17.06, 32.25 | 21.97, 23.61 |
|  | **WB BMC (g)** | 62 | 2240.88 (304.63) | 1750.51, 3002.37 | 2163.52, 2318.24 |
|  | **Head BMC (g)** | 62 | 442.96 (58.05) | 312.74, 607.42 | 428.22, 457.71 |
|  | **SUB BMC (g)** | 62 | 1799.53 (273.72) | 1325.07, 2497.66 | 1730.02, 1867.04 |
|  | **Head:WB BMC** | 62 | 0.20 (0.02) | 0.160, 0.250 | 0.193, 0.205 |
| **6** | **Chron. Age (yrs)** | 45 | 19.46 (1.25) | 15.86, 23.18 | 19.08, 19.83 |
|  | **Weight (kg)** | 45 | 59.74 (10.96) | 44.00, 101.80 | 56.45, 65.04 |
|  | **Height (cm)** | 45 | 163.48 (6.83) | 145.50, 178.50 | 161.42, 165.53 |
|  | **BMI (kg/m^2^)** | 45 | 22.31 (3.42) | 17.34, 34.05 | 21.28, 23.33 |
|  | **WB BMC (g)** | 45 | 2255.48 (309.31) | 1720.19, 3100.37 | 2162.56, 2348.41 |
|  | **Head BMC (g)** | 45 | 461.09 (60.70) | 346.88, 657.13 | 442.85, 479.33 |
|  | **SUB BMC (g)** | 45 | 1794.79 (274.07) | 1365.74, 2484.82 | 1712.45, 1877.13 |
|  | **Head:WB BMC** | 45 | 0.21 (0.02) | 0.16, 0.26 | 0.199, 0.212 |
| **8** | **Chron. Age (yrs)** | 24 | 21.71 (1.25) | 19.33, 25.24 | 21.18, 22.24 |
|  | **Weight (kg)** | 24 | 56.43 (5.87) | 43.20, 68.40 | 53.96, 58.91 |
|  | **Height (cm)** | 24 | 162.10 (7.43) | 146.40, 172.60 | 158.96, 165.24 |
|  | **BMI (kg/m^2^)** | 24 | 21.49 (2.04) | 18.90 (27.80) | 20.63, 22.36 |
|  | **WB BMC (g)** | 24 | 2184.08 (228.10) | 1798.02, 2561.98 | 2087.76, 2280.41 |
|  | **Head BMC (g)** | 24 | 463.16 (58.89) | 380.36, 567.79 | 438.29, 488.02 |
|  | **SUB BMC (g)** | 24 | 1720.88 (201.07) | 1.98.74, 2125.53 | 1635.98, 1805.79 |
|  | **Head:WB BMC** | 24 | 0.213 (0.02) | 0.16, 0.26 | 0.203, 0.222 |
| **10** | **Chron. Age (yrs)** | 16 | 23.69 (1.56) | 21.24, 27.23 | 22.86, 24.52 |
|  | **Weight (kg)** | 16 | 55.75 (6.91) | 45.20, 68.20 | 52.07, 59.43 |
|  | **Height (cm)** | 16 | 160.81 (7.47) | 146.00, 172.40 | 156.83, 164.79 |
|  | **BMI (kg/m^2^)** | 16 | 21.52 (1.88) | 18.25, 25.26 | 20.52, 22.53 |
|  | **WB BMC (g)** | 16 | 2187.45 (264.18) | 1805.42, 2618.60 | 2046.68, 2328.22 |
|  | **Head BMC (g)** | 16 | 483.71 (64.35) | 396.86, 583.21 | 449.42, 517.99 |
|  | **SUB BMC (g)** | 16 | 1703.97 (216.18) | 1393.47, 2094.86 | 1588.78, 1819.16 |
|  | **Head:WB BMC** | 16 | 0.22 (0.02) | 0.19, 0.26 | 0.212, 0.231 |
| **12** | **Chron. Age (yrs)** | 11 | 25.46 (1.26) | 23.33, 27.09 | 24.61, 26.31 |
|  | **Weight (kg)** | 11 | 59.07 (9.13) | 46.0, 68.8 | 52.94, 65.21 |
|  | **Height (cm)** | 11 | 159.99 (9.02) | 146.90, 171.90 | 153.93, 166.05 |
|  | **BMI (kg/m^2^)** | 10 | 23.38 (3.33) | 18.01, 29.09 | 20.99, 25.76 |
|  | **WB BMC (g)** | 11 | 2175.85 (270.36) | 1846.01, 2587.97 | 1994.22, 2357.48 |
|  | **Head BMC (g)** | 11 | 483.92 (63.10) | 398.72, 589.05 | 441.53, 526.30 |
|  | **SUB BMC (g)** | 11 | 1691.93 (233.46) | 1425.60, 2083.54 | 1535.09, 1848.77 |
|  | **Head:WB BMC** | 11 | 0.22 (0.02) | 0.19, 0.27 | 0.208, 0.239 |
| Chron. Age = Chronological Age; BMI= body mass index; BMC= bone mineral content; WB BMC= whole body bone mineral content; SUB BMC = sub-cranial BMC (total body, less head); sd= standard deviation | | | | | |
